# Genome sequence of *Pedobacter glucosidilyticus* DD6b, isolated from zooplankton *Daphnia magna*

**DOI:** 10.1186/s40793-015-0086-x

**Published:** 2015-11-11

**Authors:** Anja Poehlein, Rolf Daniel, Diliana D. Simeonova

**Affiliations:** Genomic and Applied Microbiology and Göttingen Genomics Laboratory, Georg-August University Göttingen, D-37077 Göttingen, Germany; Laboratory of Microbial Ecology, Department of Biology, University of Konstanz, Universitaetsstr. 10, D-78457 Konstanz, Germany; Current address: Laboratory of Microbial Biochemistry, Department of General Microbiology, Institute of Microbiology, Bulgarian Academy of Sciences, 26 Georgi Bonchev str., 1113 Sofia, Bulgaria

**Keywords:** *Pedobacter glucosidilyticus*, Phosphite assimilation, *Sphingobacteriia*

## Abstract

The phosphite assimilating bacterium, *P. glucosidilyticus* DD6b, was isolated from the gut of the zooplankton *Daphnia magna.* Its 3,872,381 bp high-quality draft genome is arranged into 93 contigs containing 3311 predicted protein-coding and 41 RNA-encoding genes. This genome report presents the specific properties and common features of *P. glucosidilyticus* DD6b genome in comparison with the genomes of *P. glucosidilyticus* type strain DSM 23,534, and another five *Pedobacter* type strains with publicly available completely sequenced genomes. Here, we present the first journal report on *P. glucosidilyticus* genome sequence and provide information on a new specific physiological determinant of *P. glucosidilyticus* species.

**Electronic supplementary material:**

The online version of this article (doi:10.1186/s40793-015-0086-x) contains supplementary material, which is available to authorized users.

## Introduction

*Pedobacter glucosidilyticus* strain DD6b was isolated from the crustacean *Daphnia magna* gut microbial community. During a study on nutritional needs of *D. magna*, the gut microbiota was investigated over time, under starvation stress and after host death [1, 2].

*Daphnia* spp. are small members of the zooplankton and key organisms in freshwater food webs. Heterotrophic bacteria contribute significantly to the nutrition of *Daphnia* species [3, 4] and are often characterized by high P:C values [5], indicating that they are a rich source of phosphorus for zooplankton [6].

Furthermore, some bacteria can assimilate reduced inorganic and organic P compounds (phosphite [+III] and organophosphonates, [7–15]) under phosphate starvation. Organophosphonates have been found in a variety of biologically produced molecules including antibiotics, phosphonolipids, phosphonoproteins, phosphonoglycans [7]. The most common naturally occurring phosphonate 2-AEP or ciliatine serves as a precursor in the biosynthesis of phosphonolipids in marine single celled organisms, sea anemones or ciliated protozoan. Recently, a sphingophosphonolipid was found in *Bacteriovorax stolpii*, a facultative predator that parasitizes larger Gram-negative bacteria [16].

Tests with newly isolated *P. glucosidilyticus* DD6b revealed growth with phosphite as a single P-source. This ability of the strain DD6b, together with the lack of information in the literature concerning phosphite or organophosphonate assimilation abilities of the other members of genus *Pedobacter* was the reason to investigate the genome of *P. glucosidilyticus* DD6b.

## Organism information

### Classification and features

*P. glucosidilyticus* strain DD6b is an aerobic, Gram negative, non-spore-forming and rod-shaped gliding bacterium, isolated from homogenized guts of the crustacean *Daphnia magna*. Strain DD6b is mesophilic to psychrotolerant, chemoheterotrophic and assimilates phosphite as sole P-source under phosphate starvation.

The type strain of *Pedobacter glucosidilyticus* 1-2^T^ (=CCTCC AB 206110^T^=KCTC 22438^T^=DSM 23,534) was isolated from a soil microbial community of a dry riverbed in the Xietongmen area (Tibet, China) in 2010 by Luo *et al*., [17].

**Fig. 1:**
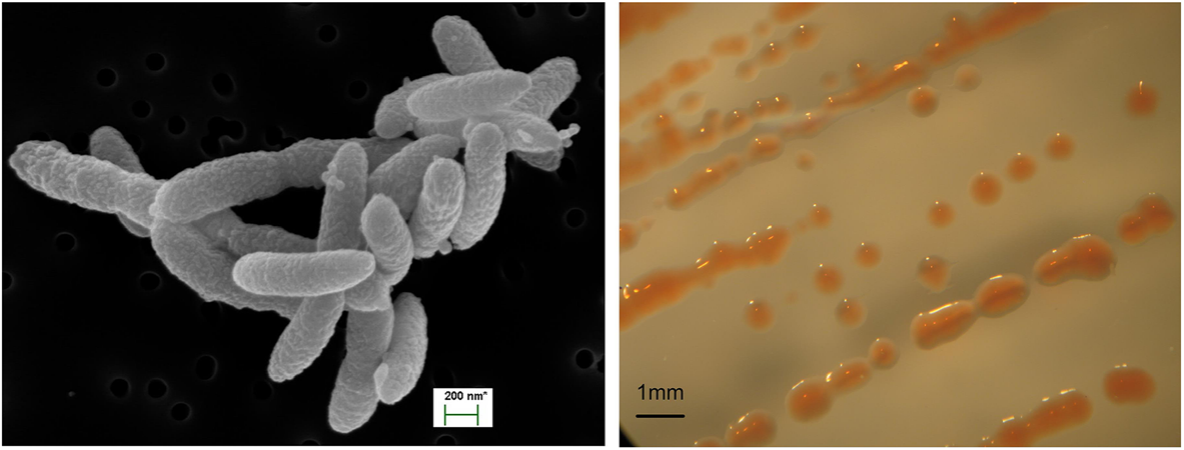
Scanning electron micrograph of *P. glucosidilyticus* strain DD6b (*Left*) and colony morphology on solid medium (*Right*)

The cells of *P. glucosidilyticus* strain DD6b are non-flagellated, non-spore-forming, flexible gliding rods with slightly rounded or tapered ends. They have protruded surfaces, and vary in size ranging from 1.0–1.2 μm in length and 0.2–0.3 μm in width (Fig. 1). Colonies (0.8–1 mm in diameter) appear after 6–7 days. They have orange-pink color on nutrient agar at 25 °C (Fig. 1, Right). Strain DD6b exhibits moderate growth, with a doubling time of 15–20 h, when grown on complex media such as nutrient broth. On chemically defined minimal medium MDS3 the strain had a doubling time of a) 7.5 h with phosphate and b) 20–23 h with phosphite as single P-source. Growth occurred at 15–28 °C, pH 7.0–7.2, and 0.2–0.5 % NaCl in the medium. Strain DD6b is motile via gliding.

*P. glucosidilyticus* strain DD6b differs slightly from the type strain of *P.glucosidilyticus *DSM 23,534 showing a weak oxidation of amygdalin, arbutin, cellobiose, lactose, methyl-α-D-mannopyranoside, methyl-α-D-glucopyranoside and salicin [17]. In addition, strain DD6b cannot oxidize glycerol or melibiose coupled with production of acids whereas for the type strain *P. glucosidilyticus *DSM 23,534 weak growth on both substrates was reported [17].

The ability of *P. glucosidilyticus* strain DD6b to grow on phosphite was proved by cultivating it successfully after 3 successive transfers on MDS3 medium, supplemented with 1 mM phosphite as sole P-source. The concentration of phosphite in the medium was monitored during the growth experiments as described previously [10].

Current taxonomic classification and general features of *P. glucosidilyticus* DD6b according to the minimum information about the genome sequence requirements are provided in Table 1. Additional information about the genome is available in the associated MIGS record table (Additional file 1: Table S1).

**Table 1:** Classification and general features of *P. glucosidilyticus* strain DD6b [22]

MIGS ID	Property	Term	Evidence code^a^
	Classification	Domain *Bacteria*	TAS [39]
		Phylum	TAS [40]
		Class *Sphingobacteriia*	TAS [41]
		Order *Sphingobacteriales*	TAS [42]
		Family *Sphingobacteriaceae*	TAS [43]
		Genus *Pedobacter*	TAS [43, 44]
		Species *Pedobacter glucosidilyticus*	TAS [17]
		strain: DD6b	
	Gram stain	negative	TAS [17]
	Cell shape	Rods	IDA,TAS [17]
	Motility	Gliding, non-flagelated	IDA
	Sporulation	Non-sporulating	TAS [17]
	Temperature range	15–28 °C	IDA
	Optimum temperature	25 °C	IDA
	pH range; Optimum	6.5–7.5; 7.0	IDA
	Carbon source	glucose	IDA
MIGS-6	Habitat	gut of *D. magna*	TAS [1]
MIGS-6.3	Salinity	0.2–0.5 % NaCl (w/v)	IDA
MIGS-22	Oxygen requirement	Aerobic	IDA
MIGS-15	Biotic relationship	commensal	TAS [1]
MIGS-14	Pathogenicity	non-pathogen	NAS
MIGS-4	Geographic location	Germany/Constance	TAS [1]
MIGS-5	Sample collection	October 2008	NAS
MIGS-4.1	Latitude	47.689081	NAS
MIGS-4.2	Longitude	9.187099	NAS
MIGS-4.4	Altitude	405 m a.s.l.	NAS

^a^Evidence codes - *IDA* Inferred from Direct Assay, *TAS* Traceable Author Statement (i.e., a direct report exists in the literature), *NAS* Non-traceable Author Statement (i.e., not directly observed for the living, isolated sample, but based on a generally accepted property for the species, or anecdotal evidence). These evidence codes are from the Gene Ontology project [45]

**Fig. 2:**
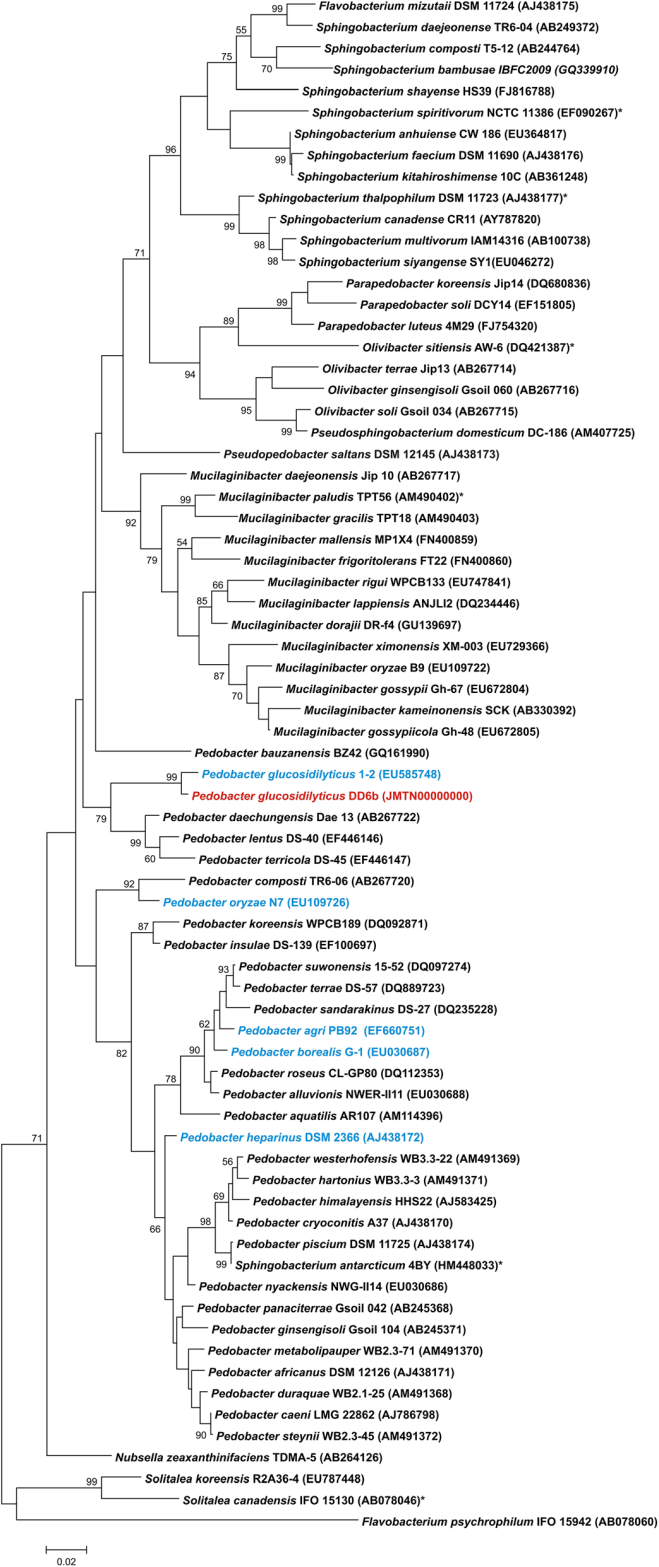
Phylogenetic tree based on 16S rRNA gene sequences of all types trains of *Sphingobacteriaceae*: The dendrogram was calculated with MEGA5 [18] using the Maximum Likelihood method based on the Jukes-Cantor model [19]. The analyzed sequences were aligned by CLUSTALW [21]. The clustering of the sequences was tested by the bootstrap approach with 1000 resamplings. The length of the tree branches was scaled according to the number of substitutions per site (see size bar). *P. glucosidilyticus* DD6b is marked in red, *Pedobacter* species with publicly available genome sequences are marked in blue. All other genome-sequenced species are marked with an asterisk

The phylogenetic neighborhood of *P. glucosidilyticus* DD6b based on 16S rRNA sequence is shown in Fig. 2. A comparison of 16S rRNA of *P. glucosidilyticus* strain DD6b with the non-redundant nucleotide collection of NCBI using MegaBlast revealed 98 % sequence identity to 16S rRNA gene sequences of uncultured *Pedobacter* sp. clone BF 061 (1461/1484 bps; NCBI accession: KC994741) and *Pedobacter glucosidilyticus* strain HME8545 (1378/1399 bps; NCBI accession: KC157040), respectively.

The phylogenetic tree was produced with MEGA5 [18] using the Maximum Likelihood method based on the Jukes Cantor model [19]. Sequences of all *Sphingobacteriaceae* type strains were downloaded from RDP [20], aligned by CLUSTALW [21] and tested by the bootstrap approach with 1000 resamplings. The length of the tree branches was scaled according the number of substitutions per site. Strain DD6b clustered clearly within the *Pedobacter* group and next to *P. glucosidilyticus* 1-2^T^.

## Genome sequencing information

### Genome project history

This organism was selected for sequencing on the basis of its environmental relevance to issues in global P cycle and the source of isolation. The genome project is deposited in GenBank database (JMTN00000000) and an improved high-quality-draft genome sequence in IMG (2590828803). Genome sequencing and annotation were done at Göttingen Genomics Laboratory (Georg-August-University Göttingen), while cultivation and analysis were performed at the University of Konstanz. A summary of the project information and its association with MIGS version 2.0 compliance [22] is shown in Table 2.

**Table 2:** Project information

MIGS ID	Property	Term
MIGS 31	Finishing quality	Improved-high-quality draft
MIGS-28	Libraries used	Illumina paired-end library; Nextera XT
MIGS 29	Sequencing platforms	Illumina XT
MIGS 31.2	Fold coverage	120 × Illumina
MIGS 30	Assemblers	SPAdes
MIGS 32	Gene calling method	Prodigal
	Locus Tag	PBAC
	Genbank ID	JMTN00000000
	GenBank Date of Release	December 8^th^, 2014
	GOLD ID	Gp0043583
	BIOPROJECT	PRJNA246159
MIGS 13	Source Material Identifier	DD6b
	Project relevance	Ecology

### Growth conditions and genomic DNA preparation

*P. glucosidilyticus* DD6b was grown in nutrient broth. A newly developed chemically defined medium MDS3 was used to study carbohydrate, phosphite and phosphate assimilation. Phosphite and phosphate assimilation tests were performed in MDS3 medium supplemented with 0.1 to 1 mM phosphite or phosphate as single P- source. Glucose 10 mM final concentration was used as C- source. The chemical composition and preparation of MDS3 medium are given in Additional file 2: Data S2. The DNA extraction procedure was performed in the following way: 4 ml of a well grown fresh culture reaching its maximum optical density (OD_600nm_ = 0.291 ± 0.040) were spun down at 13 000 × *g* in a benchtop micro centrifuge for 5 min. The supernatant was discarded and the cell pellet was suspended in the cell lysis solution from the Purgene Core Kit B (Qiagen, Hilden, Germany). Further, the extraction was performed as per manufacturer’s instructions, following the protocol for Gram-negative bacteria. The genomic DNA yield was 47.7 ng/μl. The purity of the preparation was estimated with NanoDrop ND-1000 (Thermo Fisher Scientific, Germany), with an UV absorbance ratio at 260/280 nm of 2.33, and an UV absorbance ratio at 260/230 of 0.84.

### Genome sequencing and assembly

The extracted genomic DNA was used for whole genome sequencing employing a Genome Analyzer II (Illumina, San Diego, CA, USA). Shotgun libraries were prepared according to the protocol of the manufacturer. Sequencing resulted in 12,380,618 paired-end Illumina reads of 112 bp. Reads were trimmed using Trimmomatic 0.32 [23] to get rid of sequences with quality scores lower than 20 (Illumina 1.9 encoding) and remaining adaptor sequences. SPAdes 2.5 software [24] was employed for the initial *de novo* assembly and 4,150,000 reads. The final assembly resulted in 93 contigs larger than 0.5 kb from which 84 were larger than 1 kb including 68 contigs larger than 3 kb. This assembly had an average coverage of 120, N50 value of 97,360 bp and N90 value of 24,905 bp, respectively.

### Genome annotation

The software tool Prodigal [25] was used for automatic gene prediction. rRNA and tRNA genes were identified with RNAmmer and tRNAscan, respectively [26, 27]. Automatic annotation was carried out with the IMG-ER system [28, 29] and afterwards manually curated by employing BLASTP, Swiss-Prot, TrEMBL, and InterPro databases [30].

## Genome properties

The statistics of the genome are given in Table 3. The high quality draft genome was assembled into 93 contigs with a total size of 3876 Mb and an overall GC content of 34.74 mol%. A total of 3352 genes were predicted of which 3311 were protein-encoding and 41 RNAs genes (3 rRNA and 38 tRNA). Of the protein-encoding genes 2610 (77.86 %) were assigned to a putative function and the remaining 701 (20.91 %) were annotated as hypothetical proteins. The distribution of the genes into COG functional categories [31] is shown in Table 4. One CRISPR array of 46 repeats with a direct repeat length of 46 nt adjacent to an incomplete *cas* cluster comprising *cas1*, *cas2* and *cas9* was identified in the genome of *P. glucosidilyticus* DD6b*.* However, potential prophage regions were not present in the genome sequence.

**Table 3:** Genome statistics

Attribute	Value	% of Total
Genome size (bp)	3,872,381	100.00
DNA coding (bp)	3,510,386	90.65
DNA G + C (bp)	1,344,522	34.72
DNA scaffolds	93	100.00
Total genes	3352	100.00
Protein coding genes	3311	98.78
RNA genes	41	1.22
Pseudo genes	0	0
Genes in internal clusters	2465	73.54
Genes with function prediction	2610	77.86
Genes assigned to COGs	1910	73.54
Genes with Pfam domains	2646	56.98
Genes with signal peptides	482	14.38
Genes with transmembrane helices	749	22.34
CRISPR repeats	1	0

**Table 4:** Number of genes associated with general COG functional categories

Code	Value	% age	Description
J	149	7.17	Translation, ribosomal structure and biogenesis
A	0	0.00	RNA processing and modification
K	129	6.20	Transcription
L	102	4.91	Replication, recombination and repair
B	1	0.04	Chromatin structure and dynamics
D	20	0.96	Cell cycle control, Cell division, chromosome partitioning
V	35	1.68	Defense mechanisms
T	69	3.32	Signal transduction mechanisms
M	185	8.90	Cell wall/membrane biogenesis
N	4	0.19	Cell motility
U	27	1.30	Intracellular trafficking and secretion
O	76	3.66	Posttranslational modification, protein turnover, chaperones
C	123	5.92	Energy production and conversion
G	173	8.32	Carbohydrate transport and metabolism
E	154	7.41	Amino acid transport and metabolism
F	64	3.08	Nucleotide transport and metabolism
H	117	5.63	Coenzyme transport and metabolism
I	77	3.70	Lipid transport and metabolism
P	119	7.72	Inorganic ion transport and metabolism
Q	36	1.73	Secondary metabolites biosynthesis, transport and catabolism
R	255	12.27	General function prediction only
S	164	7.89	Function unknown
-	1442	43.02	Not in COGs

The total is based on the total number of protein coding genes in the genome

## Insights from the genome sequence

In the genome of *P. glucosidilyticus* DD6b two genes required for gliding motility in *Flavobacterium**jonsoniae* were identified: *gldBDFGHJ* and *gldLMN* [32, 33]. The presence of these genes indicates a gliding motility ability of *P. glucosidilyticus* DD6b, which was not reported for the *P. glucosidilyticus* type strain [17, 34–36].

**Fig. 3:**
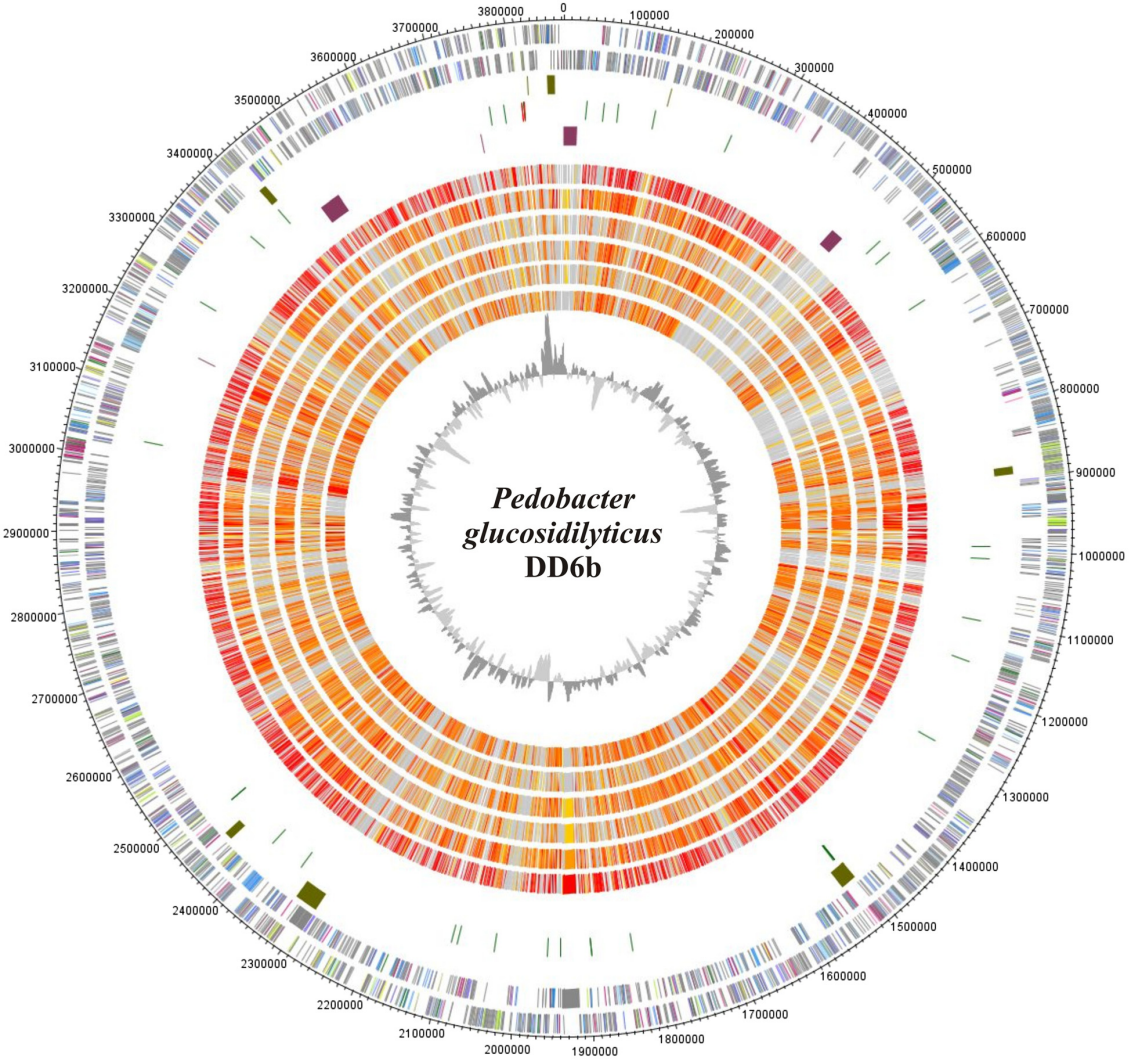
Genome comparison of *P. glucosidilyticus* DD6b with 6 completely genome-sequenced *Pedobacter* type strains: Genes encoded by the leading and the lagging strand (circle 1 and 2) of *P. glucosidilyticus* DD6b are marked in COG colors in the artificial chromosome map. Visualization was done with DNAPlotter [46]. Genomic islands (olive) identified with IslandViewer [47] are shown in circle 3, tRNAs (green) and rRNAs (pink) in circle 4. Special features of strain DD6b are marked in purple in circle 5 and described in the plain text. The presence of orthologs (circle 6 to 11) is indicated for the genomes of *P. glucosidilyticus*, DSM 23,534 (AULF00000000), *P. borealis* DSM 19,626 (JAUG00000000), *P. heparinus* HIM 762–3, DSM 2366 (CP001681.1), *P. agri PB92* (AJLG00000000), *P. oryzae* DSM 19,973 (AUHA00000000) are illustrated in red to light yellow and singletons in grey (grey: >e^−10^-1; light yellow: <e^−50^- > e^−10^; gold: <e^−50^- > e^−90^; light orange: <e^−90^- > e^−100^; orange: <e^−100^- > e^−120^; red: <e^−120^-0). The innermost plot represents the GC-content. Paralogous genes were excluded from this analysis

Another specific property of *P. glucosidilyticus* DD6b in comparison with the *P. glucosidilyticus* type strain, is the presence of a complete DNRA pathway allowing the reduction of nitrate to ammonium, where the reduction of nitrate to nitrite proceeds through an assimilatory ferredoxin-nitrate reductase *narB* (PBAC_22000) and the reduction of nitrite to ammonia by a dissimilatory nitrite reductase *nirBD* (PBAC_21900; PBAC_21910). The positions of the gene clusters for DNRA pathway and *phoPR* are shown in Fig. 3 (circle 5, clusters 1 and 2, clockwise).

Strain DD6b assimilates phosphite and most probably can assimilate phosphonoacetate under phosphate starvation to support its growth. Phosphite oxidation in strain DD6b proceeds via a sec-dependent periplasmic alkaline phosphatase (PBAC_31300), analogously to *E.coli* [15]. The genome sequence of strain DD6b suggests that this bacterium should be able to assimilate phosphonoacetate under phosphate starvation, as the gene encoding phosphonoacetate hydrolase *phnA* (PBAC_28850) catalyzing the hydrolysis of phosphonoacetate to acetate and phosphate is present. Strain DD6b does not harbor a complete 2-aminoethylphosphonate degradation pathway, despite the presence of a phosphonoacetate hydrolase encoding gene. Also, neither genes encoding carbon-phosphorus lyase complex, nor genes coding for phosphonopyruvate hydrolase or phosphonoacetaldehyde hydrolase were detected in the genome. The regulation of the phosphorus homeostasis in Gram negative bacteria is under the control of the two-component signal transduction pathway of the Pho regulon, encoded by *phoPR*. Also, 8 copies of the gene encoding alkaline phosphatase synthesis sensor protein PhoR were identified in the genome of *P. glucosidilyticus* DD6b one of which (PBAC_27380) was specific for this genome, without present orthologs in the rest of the *Pedobacter* type species whole genome sequences. The second component PhoP is present with two orthologous genes in the genome of DD6b.

In addition, a whole genome comparison of *P. glucosidilyticus* DD6b genome with the genomes of *P. glucosidilyticus *DSM 23,534 (AULF00000000), *P. borealis *DSM 19,626 (JAUG00000000), *P. heparinus* HIM762-3 DSM 2366 (CP001681), *P. agri* PB92 (AJLG00000000), *P. oryzae *DSM 19,973 ( AUHA00000000) was performed in order to define the size of the core and pan genomes of *Pedobacter* species. For this analysis Proteinortho software (blastp,[37]) with an identity cutoff of 50 %, and an E-value of 1e^10^ was used. The six compared species have a core genome of 1398 and a pan genome of 9962 orthologous groups. The highest number of orthologous groups (2387) was found for *P. glucosidilyticus* DD6b and *P. glucosidilyticus *DSM 23,534, whereas the lowest number (1675) was found for the pairwise comparison of *P. glucosidilyticus* DD6b with *P. oryzae *DSM 19,973. This genome comparison also revealed 225 specific orthologous groups only for both *P. glucosidilyticus* strains. Two of those specific orthologous CDS were a pectate lyase (PBAC_03170 and H510DRAFT_00682) and a periplasmic alkaline phosphatases, PhoD-like (PBAC_31300 and H510DRAFT_02447).

Also, *P. glucosidilyticus* DD6b has 447 singletons, the majority of which encode proteins of unknown function. Amongst the unique genes with assigned functions were found those encoding the synthesis of a green-light absorbing proteorhodopsin (PBAC_30230) playing a role in the generation of phototrophic energy (Fig. 3, position 3), proteins involved in the synthesis of EPS and capsule formation. Specific for the genome of *P. glucosidilyticus* DD6b are a rhamnogalacturonate lyase (PBAC_06010) and a second specific pectate lyase coding genes (PBAC_05900) participating in the degradation of rhamnogalacturonan I and pectin [38]. A comprehensive genome properties comparison of the 5 publicly available *Pedobacter* type strains genomes and the *P. glucosidilyticus* DD6b used in this study is presented in Additional file 3: Table S3.

## Conclusions

In this work we report on the first whole genome sequence of *P. glucosidilyticus*, strain DD6b, its specific and common genome features as a member of the genus *Pedobacter*. The whole genome comparison of *P. glucosidilyticus* DD6b with 5 other publicly available whole genome *Pedobacter* type strains sequences (as on 5th of December 2014), revealed a core genome of 1398 orthologous genes or about 30 % of each genome. The number of common orthologous groups amongst all genomes varied in the range 2387 (71.2 %) for *P. glucosidilyticus* DD6b / *P. glucosidilyticus *DSM 23,534 genome couple and 1675 (49.97 %) for *P. glucosidilyticus* DD6b/ *P. oryzae *DSM 19,973 couple. This shows relatively wide genome plasticity within the genus *Pedobacter*.

Specific for *P. glucosidilyticus* DD6b genome is the presence of genes related to phytopathogenicity and pectine degradation, as well as for assimilative nitrate reduction.

Based on physiological experiments, we proved that *P. glucosidilyticus* DD6b assimilates phosphite as single phosphorus source, in agreement with the presence of a periplasmic alkaline phosphatase-encoding gene in the genome of the strain. Furthermore, the presence of an orthologous alkaline phosphatase gene in the genome of *P. glucosidilyticus *DSM 23,534 strongly suggests that the type strain might possess this ability too. Common and specific only for both *P. glucosidilyticus* genomes was the presence of a phosphonoacetate hydrolase (PhnA) encoding gene, suggesting phosphonoacetate utilization ability for both strains. However, their genomes do not encode a complete 2-aminoethylphosphonate degradation pathway. Finally, none of the 6 analyzed *Pedobacter* genomes encoded any of the rest known organophosphonate degradation pathways. Overall members of genus *Pedobacter* species are characterized by low diversity and distribution of inorganic and organophosphonate degradation pathways. However, in future studies the phosphite assimilation property of *P. glucosidilyticus* species can be regarded as specific physiological determinant within genus *Pedobacter* (Additional file 4).

## Additional files

Additional file 1: Table S1. Associated MIGS record, *P. glucosidilyticus* DD6b (PDF 30 kb) Additional file 2: Data S2. (DOCX 17 kb) Additional file 3: Table S3. (PDF 64 kb) Additional file 4:
**The Annotation Summary; GenBank Accession Summary; Strain ID Summary; Plant Name Summary; Scientific Name Summary; Reference Search Summary** (DOC 33 kb)

## Abbreviations

2-AEP: 2-aminoethylphosphonate
