# Supplementary material for: Genome sequence of Pedobacter glucosidilyticus DD6b, isolated from zooplankton Daphnia magna
Source: Stand Genomic Sci. 2015 Nov 11;10:100. doi: 10.1186/s40793-015-0086-x (PMC4642753; doi:10.1186/s40793-015-0086-x)
Supplement: Additional file 1: Table S1. — Associated MIGS record, P. glucosidilyticus DD6b (PDF 30 kb) [file 40793_2015_86_MOESM1_ESM.pdf]

# Associated MIGS Record

**Table S1.** Associated MIGS record, *P. glucosidilyticus* DD6b

| MIGS-ID | field name | description |
|---|---|---|
| **MIGS-1** | Submit to INSDC/Trace archives | |
| **1.1** | PID | 246159 |
| **1.2** | Trace Archive | SRR1693843 |
| **MIGS-2** | MIGS CHECK LIST TYPE | |
| **MIGS-3** | Project Name | *Pedobacter glucosidilyticus* DD6b |
| **MIGS-4** | Geographic Location | Constance, Germany |
| **4.1** | Latitude | 47.689081 |
| **4.2** | Longitude | 9.187099 |
| **4.3** | Depth | Not reported |
| **4.4** | Altitude | Not reported |
| **MIGS-5** | Time of Sample collection | October, 2008 |
| **MIGS-6** | Habitat (EnvO) | Gut of *D. magna* [1] |
| **6.1** | temperature | 15-28°C |
| **6.2** | pH | 7.0 |
| **6.3** | salinity | Non-halophile, 0.2 to 0.5g.L$^{-1}$ NaCl |
| **6.4** | chlorophyll | Not reported |
| **6.5** | conductivity | Not reported |
| **6.6** | light intensity | Not reported |
| **6.7** | dissolved organic carbon (DOC) | Not reported |
| **6.8** | current | Not reported |
| **6.9** | atmospheric data | Not reported |
| **6.10** | density | Not reported |
| **6.11** | alkalinity | Not reported |
| **6.12** | dissolved oxygen | Not reported |
| **6.13** | particulate organic carbon (POC) | Not reported |
| **6.14** | phosphate | 0.1-1mM; up to 30mM |
| **6.15** | nitrate | Not reported |
| **6.16** | sulfates | Not reported |
| **6.17** | sulfides | Not reported |
| **6.18** | primary production | Not reported |
| **MIGS-7** | Subspecific genetic lineage | |
| **MIGS-9** | Number of replicons | |
| **MIGS-10** | Extrachromosomal elements | |
| **MIGS-11** | Estimated Size | |
| **MIGS-12** | Reference for biomaterial or Genome report | |
| **MIGS-13** | Source material identifiers | |
| **MIGS-14** | Known Pathogenicity | Not reported |
| **MIGS-15** | Biotic Relationship | Not reported |
| **MIGS-16** | Specific Host | *D. magna* |
| **MIGS-17** | Host specificity or range (taxid) | |
| **MIGS-18** | Health status of Host | Healthy |
| **MIGS-19** | Trophic Level | |
| **MIGS-22** | Relationship to Oxygen | Aerobe |
| **MIGS-23** | Isolation and Growth conditions | [1,2], IDA |
| **MIGS-27** | Nucleic acid preparation | |
| **MIGS-28** | Library construction | Illumina paired-end library; Nextera XT |
| **28.1** | Library size | Illumina: 1 kb |
| **28.2** | Number of reads | 12.380.618 |

| | | |
|---|---|---|
| **28.3** | vector | |
| **MIGS-29** | Sequencing method | Illumina GAII |
| **MIGS-30** | Assembly | |
| **30.1** | Assembly method | SPAdes |
| **30.2** | estimated error rate | |
| **30.3** | method of calculation | |
| **MIGS-31** | Finishing strategy | |
| **31.1** | Status | Improved high qualitity draft |
| **31.2** | coverage | 120 |
| **31.3** | contigs | 93 |
| **MIGS-32** | Relevant SOPs | |
| | Gene calling method | prodigal |
| | INSDC ID | JMTN00000000 |
| | NCBI project ID | 246159 |
| | Database: IMG | 2590828803 |
| | Genbank Date of Release | 08th Dec, 2014 |
| | GOLD ID | Gp0043583 |
| **MIGS-33** | Relevant e-resources | Data, GenBank (http://www.ncbi.nlm.nih.gov/nuccore/JMTN00000000.1) |
