# Supplementary material for: Genome sequence of Pedobacter glucosidilyticus DD6b, isolated from zooplankton Daphnia magna
Source: Stand Genomic Sci. 2015 Nov 11;10:100. doi: 10.1186/s40793-015-0086-x (PMC4642753; doi:10.1186/s40793-015-0086-x)
Supplement: Additional file 2: Data S2. — (DOCX 17 kb) [file 40793_2015_86_MOESM2_ESM.docx]

**Supplementary Data S2:**

**Composition of MDS3 medium**

The new chemically defined medium MDS3, developed to study phosphite and phosphate uptake and assimilation was prepared as follows:

20mM Tris-HCl, pH: 7.0-7.2;

Solution 1 (in g.l^-1^): MgSO_4_.7H_2_0 - 0.12; NH_4_Cl - 0.27; KCl - 0.5; NaCl - 1.0; prepared as 10x stock solution and autoclaved at 121°C for 25 min;

Solution 2: CaCl_2_.6H_2_O - 0.132g in 10 ml ddH_2_O, autoclaved at 121°C for 25 min;

SL10 Solution [[1](#_ENREF_1)]: Trace Elements Solution 10;

7 Viamine Solution [[2](#_ENREF_2)] - filter sterilized;

MDS3 preparation (1l): Tris-HCl buffer (10x stock) – 100 ml;

Solution 1 (10x stock) – 100 ml;

Solution 2 - 0,1 ml;

Trace Elements Solution 10 - 1 ml;

7 Vitamins Solution 10 - 1 ml;

q.s.p. with autoclaved doubly distilled H_2_O to 1l.

As a single phosphorus source 0.1 to 1mM phosphite or phosphate were supplemented into the MDS3. Glucose 10mM was used as a carbon source.

The medium is suitable for analogous studies with organophosphonates, since it does not include any phosphorus containing chemicals.

**References:**

1. Widdel F, Kohring G-W, Mayer F. Studies on dissimilatory sulfate-reducing bacteria that decompose fatty acids. Arch Microbiol 1983;134(4):286-294. <http://dx.doi.org/10.1007/BF00407804>

2. Widdel F, Pfennig N. Studies on dissimilatory sulfate-reducing bacteria that decompose fatty acids. I. Isolation of a new sulfate-reducer enriched with acetate from saline environments-description of *Desulfobacter postgatei* gen. nov. sp. nov. Arch Microbiol 1981;129:395-400. <http://dx.doi.org/10.1007/BF00406470>
