# Supplementary material for: Genome sequence of Pedobacter glucosidilyticus DD6b, isolated from zooplankton Daphnia magna
Source: Stand Genomic Sci. 2015 Nov 11;10:100. doi: 10.1186/s40793-015-0086-x (PMC4642753; doi:10.1186/s40793-015-0086-x)
Supplement: Additional file 3: Table S3. — (PDF 64 kb) [file 40793_2015_86_MOESM3_ESM.pdf]

Supplementary Table S3: Comparison of the genome properties of *P. glucodililyticus* DD6b with all completely sequenced type strains belonging to genus *Pedobacter* , as on 05-th of December 2014.

Genome Name	***P. agri* PB92**	***P. borealis* DSM 19626**	***P. glucosidilyticus* DD6b**	***P. glucosidilyticus* DSM 23534**	***P. heparinus* HIM 762-3, DSM 2366**	***P. oryzae* DSM 19973**
GenBank Project ID	PRJNA84275	PRJNA236461	PRJNA246159	PRJNA188803	PRJNA27949	PRJNA185613
IMG Genome ID	2548876984	2585427687	2590828803	2523533576	644736398	2523231054
GenBank Accession	AJLG00000000	JAUG00000000	JMTN00000000	AULF00000000	CP001681	AUHA00000000
Genome Size	5141552	5544917	3872381	4021020	5167383	3445768
Gene Count	5188	5008	3352	3524	4339	3122
Coding Base Count %	87.71	85.13	90.65	90.54	93.36	90.92
GC	.38	.38	.35	.34	.42	.38
COG Count	2182	2391	1910	1913	2320	1784
COG %	42.06	47.74	56.98	54.28	53.47	57.14
KEGG Count	788	866	823	812	879	760
KEGG %	15.19	17.29	24.55	23.04	20.26	24.34
KOG Count	639	717	641	636	760	633
KOG %	12.32	14.32	19.02	18.05	17.52	20.28
Pfam Count	3559	3549	2646	2749	3458	2435
Pfam %	68.6	70.87	78.94	78.01	79.7	77.99
TIGRfam Count	1091	1119	1005	1025	1156	917
TIGRfam %	34.36	22.34	29.98	29.09	26.64	29.37
Signal Peptide Count	658	729	482	448	691	371
Transmembrane Count	1108	1105	749	767	1051	774
w/ Func Pred %	65.11	66.79	77.86	52.07	66.26	75.3
Scaffold Count	379	216	93	62	1	10
rRNA Count	3	3	3	7	7	6
tRNA Count	49	51	38	41	45	37
CRISPR Count	0	0	1(46)	1(4)	0	0
