# Supplementary material for: Genome sequence of Pedobacter glucosidilyticus DD6b, isolated from zooplankton Daphnia magna
Source: Stand Genomic Sci. 2015 Nov 11;10:100. doi: 10.1186/s40793-015-0086-x (PMC4642753; doi:10.1186/s40793-015-0086-x)
Supplement: Additional file 4: — The Annotation Summary; GenBank Accession Summary; Strain ID Summary; Plant Name Summary; Scientific Name Summary; Reference Search Summary (DOC 33 kb) [file 40793_2015_86_MOESM4_ESM.doc]

**Strain ID Summary**

| Strain ID | Summary |
| --- | --- |
| DSM 19973 | Collection Code: DSM  Collection Name: Deutsche Sammlung von Mikroorganismen und Zellkulturen GmbH  Institution: DSM (Deutsche Sammlung von Mikroorganismen und Zellkulturen GmbH) - Deutschland  Strain ID: DSM 19973  External link:  <http://www.dsmz.de/catalogues/details/culture/DSM-19973.html> |
| HME8545 | Collection Code: HME  Collection Name: Haslemere Educational Museum  Institution: HME (Haslemere Educational Museum) - The United Kingdom  Strain ID: HME8545 |
| DSM 19626 | Collection Code: DSM  Collection Name: Deutsche Sammlung von Mikroorganismen und Zellkulturen GmbH  Institution: DSM (Deutsche Sammlung von Mikroorganismen und Zellkulturen GmbH) - Deutschland  Strain ID: DSM 19626  External link:  <http://www.dsmz.de/catalogues/details/culture/DSM-19626.html> |
| DSM 23534 | Collection Code: DSM  Collection Name: Deutsche Sammlung von Mikroorganismen und Zellkulturen GmbH  Institution: DSM (Deutsche Sammlung von Mikroorganismen und Zellkulturen GmbH) - Deutschland  Strain ID: DSM 23534  External link:  <http://www.dsmz.de/catalogues/details/culture/DSM-23534.html> |
| DSM 2366 | Collection Code: DSM  Collection Name: Deutsche Sammlung von Mikroorganismen und Zellkulturen GmbH  Institution: DSM (Deutsche Sammlung von Mikroorganismen und Zellkulturen GmbH) - Deutschland  Strain ID: DSM 2366  External link:  <http://www.dsmz.de/catalogues/details/culture/DSM-2366.html> |
| KCTC 22438T | Collection Code: KCTC  Collection Name: Korean Collection for Type Cultures  Institution: KCTC (Korean Collection for Type Cultures) - South Korea  Strain ID: KCTC 22438T  External link:  <http://www.brc.re.kr/English/_SearchView.aspx?sn=> |

**Plant Name Summary**

| Name | Summary |
| --- | --- |

**Reference Search Summary**

| Name | Occurence |
| --- | --- |
